# Supplementary material for: Associations between Household Solid Fuel Use, Obesity, and Cardiometabolic Health in China: A Cohort Study from 2011 to 2018
Source: Int J Environ Res Public Health. 2023 Feb 5;20(4):2826. doi: 10.3390/ijerph20042826 (PMC9956243; doi:10.3390/ijerph20042826)
Supplement: Supplementary file 1 [file ijerph-20-02826-s001.zip › ijerph-2131436-supplementary.pdf]

Table S1. HRs (95% CIs) for incident CMD by household solid fuel exposure as stratified by abdominal adiposity.

	**Cardiometabolic Disease (CMD)**					
	**Abdominal adiposity (n=2814)**			**Non-abdominal adiposity (n=3224)**		
	**Cases**	**Cases/PYs (1/10,000)**	**HRs (95%CI)**	**Cases**	**Cases/PYs (1/10,000)**	**HRs (95%CI)**
Cooking						
Household fuel						
Clean fuel	198	691.58	1.00	117	585.58	1.00
Solid fuel	372	1299.34	1.26 (1.06, 1.50)	280	1401.40	1.29 (1.03, 1.62)
Duration of solid fuel use			1.05 (1.01, 1.08)			1.09 (1.05, 1.13)
Types of household fuel use						
Clean fuel	198	691.58	1.00	117	585.59	1.00
Coal	93	324.83	1.35 (1.06, 1.72)	45	225.23	1.32 (0.94, 1.87)
Crop residue/wood	279	974.50	1.23 (1.02, 1.48)	235	1176.18	1.28 (1.02, 1.62)
Heating						
Household fuel						
Clean fuel	188	656.65	1.00	132	660.66	1.00
Solid fuel	382	1334.26	1.34 (1.13, 1.59)	265	1326.33	1.20 (0.97, 1.48)
Duration of solid fuel use			1.12 (1.07, 1.18)			1.13 (1.06, 1.20)
Types of household fuel use						
Clean fuel	188	656.65	1.00	132	660.66	1.00
Coal	223	778.90	1.45 (1.20, 1.75)	122	610.61	1.31 (1.03, 1.67)
Crop residue/wood	159	555.36	1.20 (0.97, 1.48)	143	715.72	1.11 (0.87, 1.41)

Note: CMD, cardiometabolic disease; HR, hazard ratio; CI, confidence interval; PYs, person-years. CMD include heart disease, stroke, and T2D. Multivariable models were adjusted for age, gender, marital status, education level, residence, smoking and alcohol consumption. *P* values<0.05 are bold.

Table S2. HRs (95% CIs) for incident CMM by household solid fuel exposure as stratified by abdominal adiposity.

	**CMM**					
	**Abdominal adiposity (n=2814)**			**Non-abdominal adiposity (n=3224)**		
	**Cases**	**Cases/PYs (1/10,000)**	**HRs (95%CI)**	**Cases**	**Cases/PYs (1/10,000)**	**HRs (95%CI)**
Cooking						
Household fuel						
Clean fuel	28	597.01	1.00	10	526.32	1.00
Solid fuel	53	1130.06	1.20 (0.76, 1.89)	24	1263.16	1.14 (0.54, 2.38)
Duration of solid fuel use			1.08 (1.00, 1.18)			1.09 (0.98, 1.22)
Types of household fuel use						
Clean fuel	28	597.01	1.00	10	526.32	1.00
Coal	13	277.19	1.31 (0.68, 2.50)	3	157.89	0.95 (0.26, 3.49)
Crop residue/wood	40	852.88	1.16 (0.71, 1.88)	21	1105.26	1.18 (0.55, 2.50)
Heating						
Household fuel						
Clean fuel	24	511.73	1.00	10	526.32	1.00
Solid fuel	57	1215.35	1.49 (0.92, 2.40)	24	1263.16	1.31 (0.64, 2.69)
Duration of solid fuel use			1.19 (1.05, 1.36)			1.23 (1.02, 1.49)
Types of household fuel use						
Clean fuel	24	511.73	1.00	10	526.32	1.00
Coal	33	703.62	1.62 (0.96, 2.76)	7	368.42	0.94 (0.35, 2.48)
Crop residue/wood	24	511.73	1.32 (0.75, 2.33)	17	894.74	1.59 (0.74, 3.41)

Note: CMM, cardiometabolic multimorbidity; HR, hazard ratio; CI, confidence interval; PYs, person-years. CMD include heart disease, stroke, and T2D. CMM is defined as occurring at least two of the above-mentioned diseases. Multivariable models were adjusted for age, gender, marital status, education level, residence, smoking and alcohol consumption. *P* values<0.05 are bold.

Table S3. Interactions between household solid fuel exposure and abdominal adiposity on the incident of CMD and CMM.

		**CMD**			**CMM**		
**Variable**	**Variable**	**Cases**	**Cases/PYs (1/10,000)**	**HRs (95%CI)**	**Cases**	**Cases/PYs (1/10,000)**	**HRs (95%CI)**
Cooking (solid fuel)	Abdominal adiposity						
-	-	117	240.69	1.00	10	151.75	1.00
-	+	198	407.32	1.65 (1.31, 2.08)	28	424.89	2.64 (1.27, 5.46)
+	-	280	576.01	1.25 (1.01, 1.56)	24	364.19	1.18 (0.56, 2.48)
+	+	372	765.27	2.10 (1.70, 2.60)	53	804.25	3.10 (1.56, 6.17)
Heating (solid fuel)	Abdominal adiposity						
-	-	132	271.55	1.00	10	151.75	1.00
-	+	188	386.75	1.52 (1.21, 1.90)	24	364.19	2.48 (1.20, 5.15)
+	-	265	545.16	1.18 (0.96, 1.45)	24	364.19	1.36 (0.65, 2.83)
+	+	382	785.85	2.04 (1.67, 2.50)	57	864.95	3.64 (1.87, 7.10)

Note: CMD, cardiometabolic disease; CMM, cardiometabolic multimorbidity; HR, hazard ratio; CI, confidence interval; PYs, person-years. CMD include heart disease, stroke, and T2D. CMM is defined as occurring at least two of the above-mentioned diseases. Multivariable models were adjusted for age, gender, marital status, education level, residence, smoking and alcohol consumption. *P* values<0.05 are bold.

Table S4. HRs (95% CIs) for incident CMD, CMM by household fuel exposure.

	CMD			CMM		
	**Cases**	**Cases/PYs (1/10,000)**	**HRs (95%CI)**	**Cases**	**Cases/PYs (1/10,000)**	**HRs (95%CI)**
Both clean fuel	178	366.18	1.00	16	242.79	1.00
Cook clean while heat solid fuel	137	281.84	1.30 (1.04, 1.62)	22	333.84	2.20 (1.17, 4.13)
Cook solid while heat clean fuel	142	292.12	1.25 (1.00, 1.57)	18	273.14	1.66 (0.86, 3.23)
Both solid fuel	510	1049.17	1.44 (1.21, 1.71)	59	895.30	1.71 (1.00, 2.93)

Note: CMD, cardiometabolic disease; CMM, cardiometabolic multimorbidity; HR, hazard ratio; CI, confidence interval; PYs, person-years. CMD include heart disease, stroke, and T2D. CMM is defined as occurring at least two of the above-mentioned diseases. Multivariable models were adjusted for age, gender, marital status, education level, residence, smoking and alcohol consumption. *P* values<0.05 are bold.

Table S5. HRs (95% CIs) for incident CMD, CMM by switching fuel types.

	N (%)	CMD			CMM		
		**Cases**	**Cases/PYs (1/10,000)**	**HRs (95%CI)**	**Cases**	**Cases/PYs (1/10,000)**	**HRs (95%CI)**
Cooking							
Persistent solid fuel	1881 (31.15)	367	754.99	1.00	47	713.20	1.00
Solid to clean fuel	1510 (25.01)	285	586.30	0.95 (0.82, 1.12)	30	455.24	0.78 (0.49, 1.23)
Clean to solid fuel	244 (4.04)	38	78.17	0.76 (0.54, 1.06)	4	60.70	0.64 (0.23, 1.79)
Persistent clean fuel	1735 (28.73)	277	569.84	0.80 (0.68, 0.94)	34	515.93	0.82 (0.52, 1.31)
Heating							
Persistent solid fuel	190 (3.15)	55	113.15	1.00	9	136.57	1.00
Solid to clean fuel	3130 (51.84)	592	1217.86	0.60 (0.46, 0.79)	72	1092.56	0.47 (0.23, 0.95)
Clean to solid fuel	6 (0.10)	1	-	-	0	-	-
Persistent clean fuel	2044 (33.85)	319	656.24	0.48 (0.36, 0.64)	34	515.93	0.35 (0.17, 0.72)

Note: CMD, cardiometabolic disease; CMM, cardiometabolic multimorbidity; HR, hazard ratio; CI, confidence interval; PYs, person-years. CMD include heart disease, stroke, and T2D. CMM is defined as occurring at least two of the above-mentioned diseases. Multivariable models were adjusted for age, gender, marital status, education level, residence, smoking and alcohol consumption. *P* values<0.05 are bold.

Table S6. HRs (95% CIs) for incident heart disease, stroke, T2D by household solid fuel exposure.

	**Heart Disease**			**Stroke**			**T2D**		
	**Cases**	**Cases/PYs (1/10,000)**	**HRs (95%CI)**	**Cases**	**Cases/PYs (1/10,000)**	**HRs (95%CI)**	**Cases**	**Cases/PYs (1/10,000)**	**HRs (95%CI)**
Cooking									
Household fuel									
Clean fuel	155	648.54	1.00	91	615.28	1.00	112	658.05	1.00
Solid fuel	317	1326.36	1.20 (0.98, 1.46)	204	1379.31	1.28 (0.99, 1.65)	212	1245.59	1.20 (0.94, 1.52)
Duration of solid fuel use			1.07 (1.03, 1.10)			1.07 (1.03, 1.12)			1.03 (0.99, 1.07)
Types of household fuel use									
Clean fuel	155	648.54	1.00	91	615.28	1.00	112	658.05	1.00
Coal	70	292.89	1.38 (1.04, 1.83)	44	297.50	1.47 (1.03, 2.11)	41	240.89	1.15 (0.81, 1.64)
Crop residue/wood	247	1033.47	1.15 (0.93, 1.41)	160	1081.81	1.22 (0.94, 1.60)	171	1004.70	1.21 (0.94, 1.56)
Heating									
Household fuel									
Clean fuel	139	581.59	1.00	94	635.56	1.00	123	722.68	1.00
Solid fuel	333	1393.31	1.50 (1.23, 1.82)	201	1359.03	1.29 (1.00, 1.65)	201	1180.96	1.03 (0.82, 1.29)
Duration of solid fuel use			1.18 (1.12, 1.25)			1.14 (1.07, 1.22)			1.05 (0.98, 1.13)
Types of household fuel use									
Clean fuel	139	581.59	1.00	94	635.56	1.00	123	722.68	1.00
Coal	180	753.14	1.70 (1.36, 2.11)	113	764.03	1.55 (1.18, 2.04)	97	569.92	1.02 (0.78, 1.33)
Crop residue/wood	153	640.17	1.30 (1.03, 1.64)	88	595.00	1.04 (0.78, 1.40)	104	611.05	1.05 (0.80, 1.37)

Note: HR, hazard ratio; CI, confidence interval; PYs, person-years. CMD include heart disease, stroke, and T2D. Multivariable models were adjusted for age, gender, marital status, education level, residence, smoking and alcohol consumption. *P* values<0.05 are bold.
